# Response to the letter to the editor: Epicardial, visceral and subcutaneous adipose tissue in heart failure with preserved ejection fraction

**DOI:** 10.1093/eschf/xvag168

**Published:** 2026-06-25

**Authors:** Michelle Lobeek, Dirk J van Veldhuisen, Michiel Rienstra, Thomas M Gorter

**Affiliations:** Department of Cardiology, University Medical Centre Groningen, University of Groningen, Hanzeplein 1, PO Box 30.001, Groningen 9700 RB, The Netherlands; Department of Cardiology, University Medical Centre Groningen, University of Groningen, Hanzeplein 1, PO Box 30.001, Groningen 9700 RB, The Netherlands; Department of Cardiology, University Medical Centre Groningen, University of Groningen, Hanzeplein 1, PO Box 30.001, Groningen 9700 RB, The Netherlands; Department of Cardiology, University Medical Centre Groningen, University of Groningen, Hanzeplein 1, PO Box 30.001, Groningen 9700 RB, The Netherlands

**Keywords:** HFpEF, Body composition imaging, Epicardial adipose tissue, Visceral adipose tissue

Dear Editor,

We would like to thank Dr Raza and colleagues for their interest in our work and for their thoughtful comments on our paper describing the association between epicardial, visceral, and subcutaneous adipose tissue (AT).^1^ Their letter further contributes to the academic discussion on this important topic.

We appreciate the recognition of the clinical relevance of AT distribution and agree with the limitations they highlight. As noted, the use of different imaging modalities for the assessment of AT depots may introduce variability. Ideally, all depots would be assessed using a single imaging technique or through calibrated approaches. Nevertheless, few studies to date directly compare these depots within the same patient cohort, and we believe our findings provide valuable insights despite this methodological limitation.

We also acknowledge the importance of functional assessment of AT, such as inflammation, fibrosis, and metabolic activity, as these features may more closely reflect the biological contribution of AT depots to heart failure with preserved ejection fraction (HFpEF) than volume alone. In our colleagues’ review, the differences in biological characteristics of various AT depots are well described, providing valuable mechanistic insights into the observed heterogeneity in cardiovascular risk.^2^ Moreover, given the substantial sex differences in AT distribution, it is feasible that sex also contributes to variations in the pathophysiology of cardiovascular diseases.^3^

We further agree that longitudinal data with clinical outcomes would strengthen the translational impact of future research, although this was beyond the scope of the present study.

The present study contributes to a better understanding of the impact of different AT depots throughout the body in the context of cardiovascular disease and underscores the importance of looking beyond body mass index. With the rapid advancement of imaging technologies and artificial intelligence assisted software, future studies may enable detailed assessment of total body composition, including muscle mass, the presence of sarcopenia and fatty infiltration. Ultimately, such developments could enable the creation of individualized cardiovascular risk profiles that integrate all relevant aspects of body composition including the metabolic contribution of the different AT and muscle depots.

In conclusion, we thank Dr Raza et al. for their constructive feedback. We hope that our study, together with their comments, will help stimulate further academic discussion on the role of AT in HFpEF.
